# Folate levels measured by LC–MS/MS in patients with colorectal cancer treated with different leucovorin dosages

**DOI:** 10.1007/s00280-014-2591-9

**Published:** 2014-09-20

**Authors:** Helena Taflin, Yvonne Wettergren, Elisabeth Odin, Kristoffer Derwinger

**Affiliations:** Department of Surgery, Institute of Clinical Sciences, The Sahlgrenska Academy at University of Gothenburg, The Sahlgrenska University Hospital/Östra, 41685 Gōteborg, Sweden

**Keywords:** Colorectal cancer, Leucovorin, 5-FU-based treatment, Methylenetetrahydrofolate, Tetrahydrofolate, Formyltetrahydrofolate

## Abstract

**Purpose:**

Calcium folinate (leucovorin), which is converted in vivo into biologically active folate, enhances the potency of 5-fluorouracil (5-FU)-based chemotherapy in colorectal cancer. A common dosage of leucovorin in adjuvant and palliative settings is 60 mg/m^2^. The aim was to determine the levels of tetrahydrofolate (THF), 5,10-methylenetetrahydrofolate (methyleneTHF), and 5-methyltetrahydrofolate (methylTHF) in tumour and mucosa of colorectal cancer patients who received different dosages of leucovorin intravenously at time of surgery.

**Methods:**

Eighty patients scheduled for colorectal resection with indication of colorectal cancer were randomised into four groups to receive leucovorin at 0, 60, 200, or 500 mg/m^2^, respectively. Blood samples were taken 10 and 30 min after leucovorin administration. Biopsy samples from tumour and mucosa were collected and snap-frozen at surgery. The levels of THF, methyleneTHF, and methylTHF in tumour and mucosa were assessed by liquid chromatography electrospray ionisation tandem mass spectrometry (LC–MS/MS) and the results were related to clinical diagnosis and therapeutic regimens.

**Results:**

The folate levels in tissue revealed extensive inter-individual variability. The mean methyleneTHF value for the four treatment groups were 880, 1,769, 3,024 and 3,723 pmol/g_ww_. Only half of the patients who received 60 mg/m^2^ leucovorin had higher levels of methyleneTHF in tumour than patients who received 0 mg/m^2^ leucovorin. Rectal cancer patients had significantly lower levels of methyleneTHF compared with colon cancer patients.

**Conclusions:**

There was a large inter-patient variability of tissue folate levels in colorectal cancer patients after supplementation with leucovorin at standardised dosage. High leucovorin doses were needed to exceed baseline methyleneTHF values, especially in rectal cancer patients. The results indicate that the standardised leucovorin dose may be insufficient to attain the full antitumour effect of 5-FU. Further studies are needed to establish whether higher dosage yields a better treatment response.

## Introduction

In 2012, colorectal cancer was the second most diagnosed cancer in Europe after breast cancer. Colorectal cancer was also responsible for the second highest number of cancer-related deaths after lung cancer [1]. Currently, the only curative therapy for patients with colorectal cancer is surgery with radical removal of the tumour. When the cancer is limited to the bowel wall, surgery itself normally attains the desired oncologic outcome. In cases of lymph node involvement, i.e. stage III disease, there is a high risk of tumour recurrence. It has been confirmed in several studies that a 5-fluorouracil (5-FU)-based chemotherapy regimen improves both overall and disease-free survival for patients with stage III disease [2–4]. Considering the numbers of patients and administered treatments, it is of great importance to find methods to tailor or at least optimise the therapy. However, even patients with high-risk profiles, such as poorly differentiated tumours without lymph node metastasis, could benefit from additional treatment [5] [6]. In this post-operative setting, the term adjuvant treatment is commonly used. 5-FU-based chemotherapy has also been shown to prolong overall survival in palliative settings, i.e. for patients with confirmed distant metastasis [7].

5-Fluorouracil was developed in the 1950s by Charles Heidelberger, who discovered that rat hepatomas were consuming the pyrimidine uracil more rapidly than normal rat liver tissue [8]. Thus, uracil was identified as a target molecule for chemotherapy. 5-FU is an analogue of uracil in which the hydrogen at position 5 is replaced by fluorine. Using the same mechanism to enter the cell as uracil, the 5-FU molecule is converted into the active metabolite 5-fluoro-2′-deoxyuridine 5′-monophosphate (FdUMP), which forms an inhibitory ternary complex with thymidylate synthase (TS) and 5,10-methylenetetrahydrofolate (methyleneTHF). This results in the inhibition of thymidylate synthesis and impairment of both DNA synthesis and DNA repair. The greatest impact is on cells that are rapidly dividing, such as tumour epithelial cells.

The response rate of colorectal tumours to 5-FU monotherapy is only around 10 %. By adding the stable calcium salt of 5-formyltetrahydrofolic acid (Calciumfolinate), which is converted in the liver into methyleneTHF, the tumour response rate can be improved to 21 %, as has been shown in a meta-analysis [9]. The Nordic FLV therapy, which is a combination of 5-FU and leucovorin (REF 12) that was introduced in the 1990s, is still the cornerstone of both adjuvant and palliative treatments for colorectal cancer in Nordic countries. The standard dosage is 500 mg/m^2^ 5-FU plus 60 mg/m^2^ leucovorin in the form of calciumfolinate, administered as an intravenous infusion 2 days in a row. The 2-day treatment is followed by a pause for 12 days. The standard protocol is usually 6 months long. The regimen has been duly updated with the incorporation of novel drugs, such as oxaliplatin and antibodies into more effective combination therapies. Although it is a well established regimen, the evidence for the leucovorin dosage used is rather limited. Different regimens used in clinical practice worldwide apply levels of leucovorin that range from 20 to 500 mg/m^2^.

Leucovorin has no intrinsic antitumour effect but it enhances the effect of 5-FU by providing the cofactor methyleneTHF in abundance and by stabilising the ternary complex [10]. However, leucovorin must first be converted in two steps into methyleneTHF, which is the active metabolite. This requirement for metabolic activation may result in inter-individual differences in uptake, thereby compromising the benefit gained from the addition of leucovorin in some of the patients. The leucovorin metabolism pathway has been described by Priest and colleagues [11].

The impressive advances that have been made in genetics and metabolite measurements (metabolomics) provide new possibilities for advanced studies of the folate metabolism and facilitate a better understanding of related cellular mechanisms. Our research group presented in 2012 an LC–MS/MS method that is sufficiently sensitive to separate and quantify different forms of folate [12]. Thus, the actual concentration of methyleneTHF, and not only of total folates, can now be measured in tissue samples. As evidenced by our recent findings, using the novel method, there is a significant variability in the folate levels in tumour and mucosa tissues between patients [13].

The aim of the present study was to determine the levels of different folate forms in tumour and mucosa tissue of patients with colorectal cancer who received different dosages of leucovorin intravenously at the time of surgery. The folate levels were related to the clinical diagnosis and therapeutic regimens used.

## Patients and methods

The study was approved by the Regional Ethics Committee in Gothenburg (EPN). Eighty patients scheduled for a colorectal resection with a cancer indication were enrolled in the study between January 2011 and January 2012. All patients gave their written informed consent. The pre-operative exclusion criteria were patient inability to understand the study information or inability to provide true informed consent. There were no other exclusion criteria (such as ASA-class, renal function or pre-operative tumour stage). The patients were pre-operatively randomised into four groups; the first served as control group and received no leucovorin. Groups 2, 3, and 4 received 60, 200, and 500 mg/m^2^ leucovorin, respectively, administered intravenously at the initiation of general anaesthesia. The leucovorin was manufactured in the form of calcium folinate (dl-leucovorin) supported by Teva Sweden AB Helsingborg. The surgeon was blinded to the dosage given. The patients were otherwise treated in accordance with normal routines and guidelines.

During surgery, at the time of removal of the surgical specimen, the research nurse collected fresh tissue samples from both the tumour and macroscopically normal-appearing mucosa located 10 cm from the tumour. The biopsies were snap-frozen in liquid nitrogen and stored at −80 °C until used. The pathology department assessed the specimens and provided tumour staging data. Based on the routine pathology reports, four patients were excluded from the study because the analysis revealed a lack of adenocarcinoma tissue; two patients had an obstruction related to diverticulitis, one had a squamous epithelial cancer, and one had a non-malignant adenoma. During analysis of blood samples, we discovered that one patient in treatment group two had received a leucovorin dose that was not according to the protocol and this patient is also excluded from the study. The main assessment was of the folate levels in the mucosa and tumour tissues in relation to treatment group. Clinical and pathology data regarding diagnosis, tumour differentiation and stage, and pre-operative treatment regimen were retrieved to assess the different groups and enable a better understanding of the factors that might influence treatment responses.

### Folate analyses

A liquid chromatography electrospray ionisation tandem mass spectrometry (LC–MS/MS) method was used to evaluate the levels of the folate derivatives, tetrahydrofolate (THF), methyleneTHF, and 5-methyltetrahydrofolate (methylTHF) in tumour tissue and adjacent mucosa, separately [12].). The LC–MS/MS analyses were performed on a waters 2795 LC separation module coupled to a waters micromass Quattro Triple-Quadrupole MS system with an electrospray ionisation (ESI) source. Folates were detected and quantified using positive electrospray. The separation of folates was performed using an Atlantis dC_18_ 3 µm, 2.1*100 mm column (waters) together with the guard column Atlantis dC_18_, 3 µm, 2.1*10 mm. The mobile phase consisting of eluent A (0.1 % of acetic acid in water) and eluent B (0.1 % acetic acid in acetonitrile) was used. The extracted ions following MRM transitions were monitored at m/z 446 → 299 for THF, m/z 458 → 311 for methyleneTHF, m/z 460 → 313 for methylTHF, and m/z 459 → 312 for Tomudex (IS). On the day of sample analysis, extraction buffer was prepared containing 50 mM phosphate buffer, pH 7.0, 1 % ascorbate, and 0.1 % β-mercaptopropanol. The tissue was weighed and placed in an Eppendorf vial and a 10× volume of extraction buffer was added. Homogenisation was performed using a TissueLyzer (two disruption steps at 25 Hz for 2.5 min). Tomudex was used as an intern standard. After deconjugation, protein precipitation, centrifugation, and ultrafiltration (30 min at 21,500×*g* at 20 °C) were performed. The solution at the bottom of the test tube was used for the LC–MS/MS analysis.

Calibration graphs were constructed by plotting the peak area ratio of each compound to internal standards against concentration. The standards and samples were processed using the QuanLynx quantitative processing tool in MassLynx (Waters Corp., Milford, MA, USA). Intra-batch variability was determined by analysing tissue Q-samples at low, medium, and high concentrations on the same day. Inter-assay variability was determined by analysing low, medium, and high concentration samples on four separate days. The relative standard deviation (RSD) ranged from 2 to 7 % for all analyses, and the variability over 4 days ranged from 3 to 14 % for all analyses. The accuracy of the method was determined by estimating the recovery by adding known amounts of the standard to a sample. The average recoveries were 98, 87, and 93 % for THF, methyleneTHF, and methylTHF, respectively [12].

The levels of THF, methyleneTHF, and methylTHF in each sample were expressed as pmol/g wet-weight (pmol/g_ww_). Due to the known interconversion of methyleneTHF and THF, the sum of the concentrations of these two folates were also calculated [14].

The plasma samples were frozen, stored, and shipped at −80 °C to Charles River Laboratories, UK, where the plasma concentrations of methyleneTHF, THF, methylTHF, and formyl-THF were analysed using a validated LC–MS/MS method.

### Statistical analyses

The JMP 11.0/SAS software (SAS Institute Inc., Cary, NC, USA) was used for the statistical analyses. Nonparametric tests; Mann–Whitney/Kruskal–Wallis, and matched-pair analyses (Wilcoxon Signed Rank test) were used to examine differences between the groups. Also presented are descriptive statistics with mean or median values and measures of dispersion, as appropriate. The significance level was set at 95 %.

## Results

### Patients and treatment regimens

Based on clinical diagnosis, 38 patients had colon cancer, 37 had rectal cancer, and three patients had cancer in both the colon and rectum synchronously. The latter three patients were excluded from the statistical analyses of folate levels according to tumour location. The demographic, clinical, and pathologic data are shown in Table 1. There were no significant differences regarding age, gender, tumour location, tumour stage, tumour differentiation, or lymph status between the four groups. The mean times between administration of leucovorin and time of biopsy sampling, as well as ranges for the three groups are shown in Table 1.

**Table 1:** Clinicopathological characteristics of the colorectal cancer patients sub grouped by leucovorin dosage

Parameter	Leucovorin dosage (*n*)			
	0 mg/m^2^ [18]	60 mg/m^2^ [18]	200 mg/m^2^ [19]	500 mg/m^2^ [20]
Age (years)				
Median	73	65.6	70	75.5
Range	43–89	42–87	37–89	37–87
Sex				
Male	11	12	7	9
Female	7	6	12	11
Tumour location				
Colon	9	10	8	11
Rectum	8	8	10	8
Colon + rectum	1	0	1	1
Primary tumour stage				
1	0	1	1	3
2	7	9	9	7
3	9	8	7	8
4	4	0	2	2
Data missing	1	0	0	0
Tumour differentiation				
Well	0	0	0	0
Moderate	14	13	11	12
Poor	3	2	6	6
Mucinous	0	3	2	2
Data missing	1	0	0	0
Pre-operative radiation				
Short-term	0	6	5	2
Long-term^a^	0	0	2	1
Short + long-term^a^	0	1	7	3
Median time (min) leucovorin administration-biopsy sampling (mean range):		170 (65–285)	165 (72–457)	163 (65–555)

^a^Neoadjuvant long-term radiation/chemotherapy

As shown, no statistical differences between the times from leucovorin administration to biopsy sampling were seen. However, a difference regarding pre-operative treatment was noted; in the control group, there was no patient with rectal cancer who had been given pre-operative radiation treatment or neoadjuvant treatment. Regarding safety, the administration of the drug was associated with temporary red cheeks in one patient (given 200 mg/m^2^ leucovorin) and a short temporary hypotension reaction in one patient (given 500 mg/m^2^). These episodes were considered as adverse events. In none of the cases did the adverse reaction lead to any change in the operating procedure.

### Folate levels in tumours and mucosa

The mean levels of methyleneTHF, THF, and methylTHF were analysed in both tumour and mucosa tissues obtained from patients of each treatment group (Table 2). The mean level of each folate increased with increasing dosage of leucovorin and showed a large inter-patient variation in all treatment groups. The folate levels differed significantly between the mucosa and tumour tissues and were generally lower in the mucosa of the control group. Patients who received 60 or 200 mg/m^2^ leucovorin had significantly higher mean levels of methyleneTHF in their tumours, as compared to the levels in their mucosal samples. After treatment with 500 mg/m^2^ leucovorin, the difference in methyleneTHF level between the tumour and mucosa samples was no longer statistically significant. The same pattern was seen when the THF concentration or the sum of methyleneTHF and THF were analysed. No significant differences in the levels of methylTHF were seen between the tumour and mucosa samples in any of the treatment groups. However, in contrast to the other folates, the methylTHF level in mucosa of the control group was significantly higher compared to the level in tumour tissue.

**Table 2:** Comparison of mean ± SD folate levels in tumour and mucosa tissues of the colorectal cancer patients

Folate form	Tissue type	Leucovorin dosage (*n*)			
		0 mg/m^2^ [18]	60 mg/m^2^ [18]	200 mg/m^2^ [19]	500 mg/m^2^ [20]
THF	Tumour	136 ± 88	249 ± 97	490 ± 353	543 ± 279
	Mucosa	103 ± 59	210 ± 96	289 ± 153	455 ± 282
*P* value		0.030	0.091	<0.0001	0.12
MethyleneTHF	Tumour	880 ± 412	1,769 ± 818	3,024 ± 1,941	3,773 ± 1,425
	Mucosa	669 ± 221	1,377 ± 470	1,883 ± 471	3,062 ± 1,445
*P* value		0.0016	0.022	0.0003	0.090
MethyleneTHF + THF	Tumour	1,016 ± 475	2,018 ± 888	3,514 ± 2,109	4,266 ± 1,563
	Mucosa	772 ± 265	1,587 ± 536	2,173 ± 461	3,517 ± 1,607
*P* value		0.010	0.018	0.0002	0.070
MethylTHF	Tumour	141 ± 86	1,056 ± 348	2,544 ± 959	4,129 ± 1,293
	Mucosa	189 ± 111	1,066 ± 384	2,295 ± 501	4,095 ± 2,093
*P* value		0.0047	0.93	0.46	0.49
Leucovorin (mean/range)^a^					
10 min		Non applicable	11,377 ± 2,225	30,900 ± 6,176	93,625 ± 1,872
30 min			8,199 ± 1,238	27,684 ± 41,013	69,445 ± 9,172

Folate levels in pmol/g_ww_

μg/L plasma

*P* value by Wilcoxon Signed Rank test

^a^Concentration in plasma

### Folate levels and tumour location

**Fig. 1:**
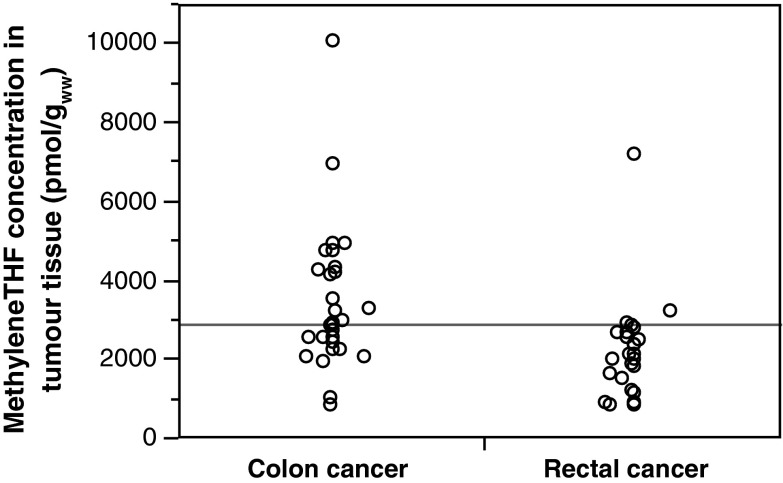
Comparison of the methyleneTHF concentration in tumour tissue of patients with colon (*n* = 29) or rectal (*n* = 28) cancer after FLV treatment. *Each circle* represents an individual patient. As shown, patients with rectal cancer had lower levels of methyleneTHF in their tumours. The *horizontal line* represents the grand mean

**Fig. 2:**
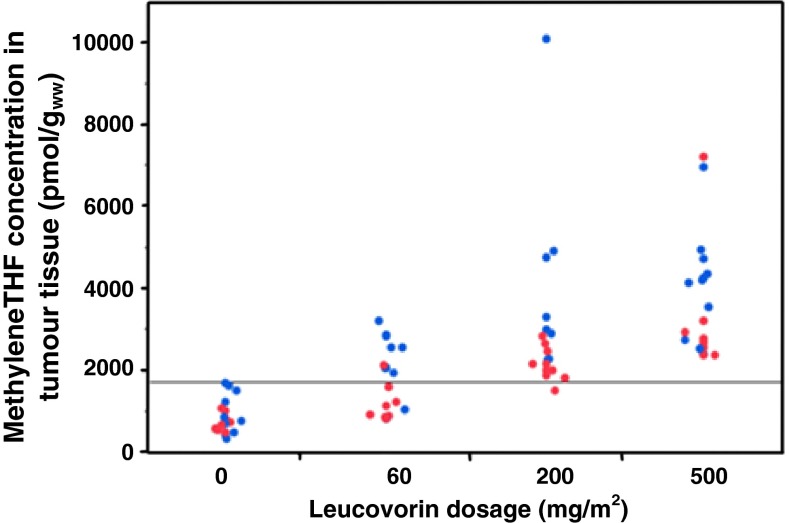
Comparison of the methyleneTHF levels in tumour tissue of patients with colon or rectal cancer after supplementation with 0, 60, 200, or 500 mg/m^2^ leucovorin in combination with 5-FU. Individual patients with colon cancer are represented by *blue dots*, rectal cancer patients with *red dots*. The *horizontal line* marks the highest methyleneTHF concentration found in the control patients (1,714 pmol/g_ww_). As shown, the tumoural methyleneTHF levels in rectal cancer patients were generally lower than in colon cancer patients after treatment with leucovorin

There were differences between the folate levels in colonic and rectal tumours according to treatment doses. For all treatments groups, the mean methyleneTHF levels in the rectal tumours were significantly lower than those in the colonic tumours (Table 3; Figs. 1, 2). The difference was significant in the groups that received 60 or 200 mg/m^2^ leucovorin. The THF level was significantly lower in rectal tumours of patients who received 60 mg/m^2^. As shown in Table 3, the methyleneTHF + THF levels were generally low in rectal, compared to colon, tumours. In contrast, the methylTHF levels were higher in rectal tumour tissues of patients who were treated with leucovorin, and a significantly higher level was seen after treatment with 60 mg/m^2^. As shown in Fig. 2, only 10 (50 %) of the patients given 60 mg/m^2^ leucovorin achieved a methyleneTHF level in their tumour tissues that was above the highest value of any patient in the control group (1,714 pmol/g_ww_). At 200 mg/m^2^ leucovorin, all patients, except one, reached methyleneTHF levels >1,714 pmol/g_ww_, and at 500 mg/m^2^ leucovorin, all patients had methyleneTHF levels above the level of the controls. Data were weighted according to time to vessel ligation, which was a parameter suspected to affect the tissue folate levels. However, this did not affect the significant differences between colon and rectal tumour tissue.

**Table 3:** Comparison of mean ± SD folate levels in tumour tissues of the colorectal cancer patients by tumour location

Folate form	Tumour location	Leucovorin dosage (*n*)			
		0 mg/m^2^ [17]	60 mg/m^2^ [18]	200 mg/m^2^ [18]	500 mg/m^2^ [19]
THF	Colon	143 ± 62	306 ± 79	508 ± 240	522 ± 164
	Rectum	139 ± 121	178 ± 67	497 ± 446	494 ± 317
*P* value		0.41	0.0067	0.18	0.24
MethyleneTHF	Colon	1,016 ± 482	2,214 ± 779	4,207 ± 2,590	4,222 ± 1,285
	Rectum	747 ± 230	1,213 ± 454	2,162 ± 406	3,222 ± 1,530
*P* value		0.26	0.024	0.0029	0.066
MethyleneTHF + THF	Colon	1,159 ± 537	2,520 ± 826	4,715 ± 2,821	4,744 ± 1,397
	Rectum	886 ± 328	1,391 ± 477	2,659 ± 696	3,716 ± 1,726
*P* value		0.26	0.0088	0.019	0.094
MethylTHF	Colon	162 ± 99	1,257 ± 201	2,331 ± 757	4,022 ± 1,551
	Rectum	123 ± 63	806 ± 338	2,795 ± 1,100	4,163 ± 1,119
*P* value		0.46	0.0067	0.56	0.54

Folate levels in pmol/g_ww_

*P* value by Kruskal–Wallis test (2-sample Test)

### Blood analysis

A strong correlation was noted between the levels of leucovorin in blood samples collected 10 and 30 min after the given dose of calcium folinate and the levels of the different folate forms in both tumour and mucosa samples. However, there was no correlation between the blood levels of leucovorin and levels of folate in tissue in relation to the administered dosage of the drug.

## Discussion

The role of leucovorin in 5-FU-based chemotherapy is to increase the level of the cofactor methyleneTHF needed to stabilise the ternary complex consisting of FdUMP, methyleneTHF, and TS in tumour cells. Thereby, the production of thymidine is inhibited leading to an impaired DNA synthesis and DNA repair. The continuous development of assessment techniques and new biochemical methods provide tools to study different metabolites in more sophisticated ways. In the present study, a sensitive LC–MS/MS method was used to analyse metabolically active folate metabolites, including methyleneTHF.

A notable finding of the study was that only 50 % of the patients who received the common dose of 60 mg/m^2^ leucovorin achieved methyleneTHF levels in the tumour tissue that were higher than those detected in patients of the control group. In contrast, in the group that received 200 mg/m^2^ leucovorin, only one patient did not reach the highest methyleneTHF level of the controls. Furthermore, in the group that received 500 mg/m^2^ leucovorin, all patients had a methyleneTHF level well above the controls. This indicates that in a large number of patients low levels of folates accumulated in the tumour, possible because of limited folate polyglutamylation. Another important finding was the significant difference in methyleneTHF and THF levels between the colorectal tumour tissue and the macroscopically normal-appearing mucosa (Table 2). This difference was observed in patients who had not received any treatment and was even more pronounced in those patients who received leucovorin at a dosage of 60 or 200 mg/m^2^.

The study raises the question whether the commonly used dosage of leucovorin might result in a sub-optimal concentration of methyleneTHF in the tumour tissue to provide an optimal effect of 5-FU treatment. If this is the case, several patients with colorectal cancer might be receiving inadequate treatment and may benefit from leucovorin concentrations as high as 200–500 mg/m^2^. Similar conclusions were drawn by Schlemmer et al. [15] in a study published in 2008, which showed significant higher values regarding reduced folates in tumour tissues as well as in liver metastasis when doses of 200 and 500 mg/m^2^ were used.

Adding to the complexity, there was a high inter-individual variation in the methyleneTHF levels in the tumours (Fig. 2). Polymorphisms in genes that code for folate-associated enzymes, such as methylenetetrahydrofolate reductase, could be a part of the explanation for this phenomenon [16–19]. Different activity of the enzymes needed in conversion of leucovorin to methyleneTHF could also play a role, as indeed could different starting levels of the tissue folates. Therefore, it is difficult to predict the levels of tissue folates that will be reached in an individual patient, in response to a given leucovorin dose based solely on the body surface area. However, since leucovorin is not considered to be a toxic substance and only a few and mild adverse events were reported in the present study, further studies with high leucovorin supplementation levels (200–500 mg/m^2^) could be reasonably pursued. Hypothetically, high doses of leucovorin would make an abundance of methyleneTHF available for ternary complex formation between methyleneTHF, FdUMP, and TS, despite possible rate-limiting steps or local folate deficiencies. The results further showed clear differences in the folate levels in relation to tumour location. The methyleneTHF and THF + methyleneTHF levels differed significantly between patients with colon and rectal cancer (Table 3). For all treatment groups, the levels were lower for rectal cancers. No significant difference was seen between patients treated or not treated with neoadjuvant therapy.

Post-operative adjuvant chemotherapy was previously less established for cases of rectal cancer than for cases of colon cancer, but is now recommended in the Swedish national guidelines in selected cases. Based on the results of our study, it appears that the administered concentration of leucovorin in the standard treatment is inadequate for patients with rectal cancer in that it yields a clinically insufficient concentration of methyleneTHF in the tumour tissue. This finding may explain why the evidence for a beneficial application of adjuvant treatment with 5-FU-based chemotherapy has not been as clear in rectal cancer as they are in colon cancer [20, 21].

The present study draws attention to several issues. The findings raise the question as to whether previous assumptions made regarding leucovorin dosages are correct. It also shows that new techniques and advances in associated scientific areas make revaluation of previously studied subjects both worthwhile and important. As the present study was limited in terms of the numbers of patients, the results needs to be confirmed in a larger study. Among the strengths of the study are the broad inclusion criteria, reflecting clinical reality, and the randomisation of the patients to the treatment groups. The randomisation should negate selection bias, although skewing was noted between patient groups in terms of neoadjuvant treatment; no patient in the control group received any pre-operative radiotherapy. Because a high turnover of folates might be required during repair of radiation-damaged tissue, the mean folate level at base line could be anticipated to be lower if the rectal cancer patients of the control group had been subjected to radiotherapy. However, we could not detect any differences in the folate levels between patients treated or not treated with radiotherapy. Furthermore, both higher (methylTHF) and lower (methyleneTHF) folate levels were found in rectal tumours as compared to colon tumours, after leucovorin supplementation. Thus, there seems to be inherent differences in the response to leucovorin treatment between rectal and colon cancer.

The possibility exists that the difference in folate levels noted is a systematic bias due to treatment or surgical issues, including differences in the time passed after vessel ligation until biopsy sampling. However, this potential confounder was tested for by including the time to vessel ligation in the statistical analysis and did not significantly affect the results. In a recent study, Sadahiro et al. showed that leucovorin administration significantly increased the reduced folate levels in colorectal cancer tissue and adjacent mucosa. The increase lasted until 18 h for mucosa and 12 h for colorectal cancer tissue [22]. However, in a clinical setting, the time span from the leucovorin administration until infusion of chemotherapy is usually no longer than 30–90 min. It can also be debated as to whether the fact that a tumour is a very heterogenic tissue has an important influence on the measured folate concentrations depending on how the biopsies are collected. In the present study, we have tried to address this problem by having the same two research nurses collecting tissues in a standardised way. The data suggest that our results are comparable to previous results reported in the literature [14, 23, 24].

A last and major challenge is the difficulty of extrapolating the findings into adjuvant treatment settings, as any visible tumour tissue has been removed by the time adjuvant treatment starts. Thus, the treatment effect will be exerted on normal tissues and, hopefully, on remaining circulating tumour cells or cell aggregations. The nature of this issue itself presents challenges that need to be resolved in order to achieve treatment improvements.

## Conclusions

The results of this study showed a large inter-patient variability of folate levels in tumour and mucosa tissue of colorectal patients after supplementation with leucovorin at the standardised dosage. The low levels of methyleneTHF were most prominent in rectal cancer patients, where high leucovorin doses were needed to exceed baseline methyleneTHF values in tumour. The results of this study indicate that the standardised dose may be sub-optimal since the achieved concentration of methyleneTHF in tissues of the patients may be insufficient to get an optimal antitumour effect of 5-FU. On going studies will establish if a higher concentration of leucovorin yields a better treatment response.
